# Supplementary material for: Carboxyl Functionalization of N-MWCNTs with Stone–Wales Defects and Possibility of HIF-1α Wave-Diffusive Delivery
Source: Int J Mol Sci. 2023 Jan 9;24(2):1296. doi: 10.3390/ijms24021296 (PMC9866222; doi:10.3390/ijms24021296)
Supplement: Supplementary file 1 [file ijms-24-01296-s001.zip › ijms-2147537-supplementary.pdf]

Supplementary Information

Carboxyl Functionalization of N-MWCNTs with Stone-Wales Defects and Possibility of HIF-1α Wave-diffusive Delivery

Vladislav V. Shunaev [1], Nadezhda G. Bobenko [2], Petr M. Korusenko [3], Valeriy E. Egorushkin [2], and Olga E. Glukhova [1,4,*]

[1] Department of Physics, Saratov State University, 410012 Saratov, Russia
[2] Laboratory of physics of nonlinear media, Institute of Strength Physics and Materials Science of SB RAS, 2/4 Academichesky Avenue, Tomsk 634021, Russia
[3] Department of Physics, Omsk State Technical University, 11 Mira Prosp., 644050 Omsk, Russia
[4] Institute for Bionic Technologies and Engineering, Sechenov University 119991 Moscow, Russia
* Correspondence: glukhovaoe@sgu.ru; Tel.: +7-8452-514562

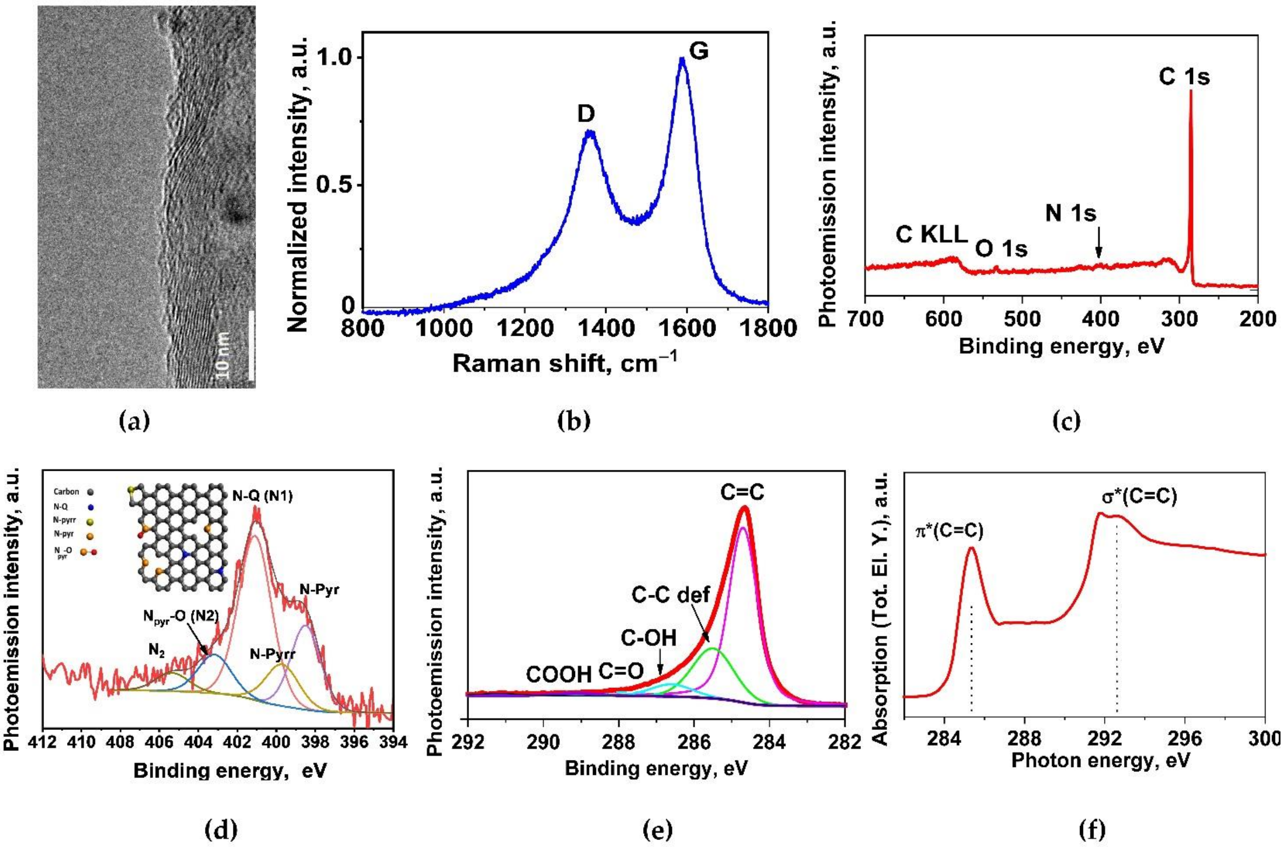

Figure S1. Experimental data on atomic and electronic structure of N-MWCNTs before irradiation: (**a**) TEM, (**b**) normalized and baseline corrected Raman spectra, (**c**) survey and (**d**) core-level N1s (N-Pyr – nitrogen in pyridinic configuration; N-Pyrr – nitrogen in pyrrolic configuration; N2 – molecular nitrogen), (**e**) C1s PE spectra of N-MWCNTs, and (**f**) their NEXAFS C1s spectrum.

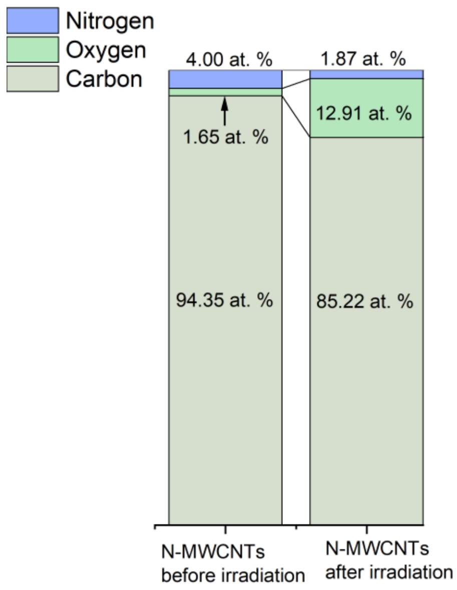

Figure S2. Diagram of atomic concentration from survey PE spectra for initial and irradiated N-MWCNTs
